# Immunonutrition – the influence of early postoperative glutamine supplementation in enteral/parenteral nutrition on immune response, wound healing and length of hospital stay in multiple trauma patients and patients after extensive surgery

**DOI:** 10.3205/iprs000074

**Published:** 2015-12-15

**Authors:** Kai J. Lorenz, Reiner Schallert, Volker Daniel

**Affiliations:** 1 Bundeswehr Hospital of Ulm, Department of Otolaryngology, Head and Neck Surgery, Ulm, Germany; 2 Bundeswehr Hospital of Ulm, Surgical Centre, Department of Accident Surgery and Orthopaedics, Ulm, Germany; 3 University of Heidelberg, Institute of Transplantation Immunology, Heidelberg, Germany

## Abstract

**Introduction:** In the postoperative phase, the prognosis of multiple trauma patients with severe brain injuries as well as of patients with extensive head and neck surgery mainly depends on protein metabolism and the prevention of septic complications. Wound healing problems can also result in markedly longer stays in the intensive care unit and general wards. As a result, the immunostimulation of patients in the postoperative phase is expected to improve their immunological and overall health.

**Patients and methods:** A study involving 15 patients with extensive ENT tumour surgery and 7 multiple-trauma patients investigated the effect of enteral glutamine supplementation on immune induction, wound healing and length of hospital stay. Half of the patients received a glutamine-supplemented diet. The control group received an isocaloric, isonitrogenous diet.

**Results:** In summary, we found that total lymphocyte counts, the percentage of activated CD4+DR+ T helper lymphocytes, the in-vitro response of lymphocytes to mitogens, as well as IL-2 plasma levels normalised faster in patients who received glutamine-supplemented diets than in patients who received isocaloric, isonitrogenous diets and that these parameters were even above normal by the end of the second postoperative week.

**Summary:** We believe that providing critically ill patients with a demand-oriented immunostimulating diet is fully justified as it reduces septic complications, accelerates wound healing, and shortens the length of ICU (intensive care unit) and general ward stays.

## Zusammenfassung

**Einleitung:** Die Prognose polytraumatisierter Patienten mit schweren Schädel-Hirntraumata sowie Patienten mit ausgedehnten Kopf-Hals-chirurgischen Eingriffen hängt in der postoperativen Phase maßgeblich vom Proteinmetabolismus sowie der Prävention von septischen Komplikationen ab. Ebenso wirken sich Wundheilungsstörungen deutlich verlängernd auf den Intensivaufenthalt und die Dauer des stationären Aufenthaltes aus. Folglich sollte sich eine immunstimulierende Versorgung der Patienten im Rahmen der postoperativen Phase verbessernd auf die Immun- und Gesamtsituation des Patienten auswirken.

**Patienten und Methoden:** In einer Studie an 15 Patienten mit ausgedehnten HNO-tumorchirurgischen Eingriffen und 7 polytraumatisierten Patienten wurden die Auswirkung einer gezielten enteralen Substitution mit Glutamin im auf die Immuninduktion, die Wundheilung sowie den stationären Aufenthalt untersucht. Die Hälfte der Patienten wurde mit einer glutaminreichen Diät ernährt, die Kontrollgruppe erhielt eine isonitrogene, isokalorische Ernährung.

**Ergebnisse:** Zusammenfassend zeigt sich, dass sich die Anzahl der Gesamtlymphozyten, der Anteil von aktivierten CD4+DR+ T-Helferlymphozyten, die In-vitro-Stimulierbarkeit der Lymphozyten mit Mitogenen sowie die IL-2 Plasmaspiegel bei glutaminreich ernährten Patienten schneller normalisieren als bei isonitrogen-isokalorisch ernähren Patienten und dass diese Parameter gegen Ende der 2. postoperativen Woche sogar „übernormal“ sind.

**Zusammenfassung:** Die gezielte frühenterale Substitution kritisch kranker Patienten mit einer bedarforientierten, immunstimulierenden Diät scheint uns unter dem Aspekt der Verringerung septischer Komplikationen, der schnelleren Wundheilung wie auch der Verkürzung des Intensiv- wie auch stationären Aufenthalts unbedingt gerechtfertigt.

## 1 Introduction

In the postoperative phase, the prognosis of multiple trauma patients after extensive surgery mainly depends on protein metabolism and the prevention of septic complications [1], [2]. Wound healing problems also result in markedly longer stays in the intensive care unit and general wards. As a result, the immunostimulation of patients in the postoperative phase is expected to improve their immunological and overall health. Clinical observations suggest that the positive effect of early nutritional therapy can be enhanced by adding certain nutrients that have a positive effect on the immune system. Pilot studies have shown that glutamine, arginine and omega-3 fatty acids have positive effects on the immune status of critically ill patients [3], [4], [5], [6], [7], [8], [9], [10], [11], [12].

Severe trauma combined with extensive surgery can lead to a variety of immunological disorders that are primarily induced by a hyperactivation and subsequent deactivation of monocytes and are characterised by loss of bone marrow function and changed regulatory mechanisms of non-specific and specific immune response.

The result is a reduction in the CD4:CD8 ratio to values <1, which can be observed more than two weeks after the injury was sustained [13].

Surgical interventions and traumas lead to a considerable inhibition of lymphocyte proliferation following stimulation by mitogens. The massive release of immunosuppressive PGE_2_ from activated macrophages plays a major role [14]. This is associated with an increase in the synthesis and secretion of proinflammatory mediators such as IL-6 and TNF-α, the concentration of which correlates with the incidence of posttraumatic infections as well as a decrease in the synthesis and secretion of, for example, IL-2, IL-3 and γ-IFN, which lowers the body’s defences even more [14], [15], [16].

With B lymphocytes, major surgery leads to a pronounced inhibition of B cell differentiation and IgM synthesis while IgA and IgG levels are normal or even increased [17].

Neutrophils, eosinophils, and monocytes/macrophages fight off bacteria by producing reactive oxygen compounds such as O_2_–, H_2_O_2_ and OCI^–^ using electrons from glucose reduction to gradually reduce molecular oxygen. This production of radicals is partially stimulated by IL-1, γ-IFN, TNF-α, TNF-β [18], [19], [20]. Oxygen radicals secreted into the environment can kill non-phagocytised bacteria but can also damage surrounding tissue; their “neutralisation” is the main reason why the body needs the glutathione system. Massive postoperative/posttraumatic oxidative stress often causes a depletion of glutathione and thus additional damage to the body by reactive oxygen compounds [21].

Since the endogenous mechanisms of the body are insufficient to ensure survival following major trauma, several therapeutic approaches to correct the pathologically changed immune response have been tested, e.g. the application of TP-5, indoniethacine, pentoxiphylline, γ-IFN, and hematopoietic growth factors. However, no generally valid, clinically relevant and statistically significant benefits have been observed [14].

The prognosis of multiple trauma patients in the postoperative phase after extensive surgery thus mainly depends on the prevention of septic complications and on protein metabolism. Wound healing problems and infectious complications lead to much longer stays in the intensive care unit and general wards.

Recently, there have been more and more indications that nutritional intervention has a positive impact on weakened immune systems. Certain nutrients, especially glutamine and antioxidative vitamins, are currently the focus of attention. Up until now, enteral (tube) feeding has been indicated only if patients have a disease- or treatment-related inability to eat for more than 10 days, if they have a functioning gastrointestinal tract, and if no contraindications are present. More recent studies [22] have shown, however, that early enteral nutrition not only makes an important contribution to maintaining the structure and function of the intestinal mucosa but also minimises the risk of bacterial translocation and resulting systemic inflammation [5], [23], [24], [25]. Studies have reported significant reductions in infectious complications (especially pneumonia) in glutamine-rich enteral control groups [11], [22], [26] and reductions, though generally not significant reductions, in the length of hospital stays [8], [12], [22], [23], [27], [28], [29], [30], [31].

The aim of this study was to examine the effect of an enteral supplementation of glutamine, arginine and omega-3 fatty acids on immune function, wound healing, and length of hospital stay in multiple-trauma patients and patients after extensive surgery. Knowledge acquired from this study may be transferred to patients with burn injuries or extensive soft tissue injuries similar to those sustained in war [3], [12], [22], [23], [32], [33], [34], [35]. The prognosis of such patients may be considerably improved by early metabolically optimised and immunomodulatory nutrition via the enteral route, which is associated with significantly fewer complications.

## 2 Current state of research

Nutrition supplementation in intensive care patients with multiple traumas or major surgery has been carried out for approximately 25 years. Initially, treatment focussed exclusively on parenteral feeding. The disadvantages of a parenteral feeding regimen include high costs and a complete avoidance of the gastrointestinal tract. This unphysiological approach to nutrition leads, within 24 hours, to villous atrophy in the small intestine and consecutive damage to the intestinal mucosa and a reduction in gastrointestinally stimulated hormones required for the metabolism of applied nutrients [22]. Susceptibility to infection is ultimately supported by the translocation of enteral intestinal flora into the bloodstream, resulting in sepsis [13]. Early enteral nutrition with standard nutrient solutions via nasogastric or nasojejunal tubes, which has been increasingly applied in recent years, can prevent villous atrophy and the increased translocation of bacteria and bacterial toxins from the intestine into the bloodstream. One disadvantage of these standard diets is, however, that their composition in terms of both basic nutrients (proteins, carbohydrates, fats) and micronutrients is not tailored to specific metabolic changes in the above-mentioned group of patients. Pilot studies involving especially glutamine, arginine and omega-3 fatty acid supplements have, however, shown promising positive effects on wound healing, immune system stimulation, and length of hospital stay [11], [13], [14], [15], [17], [22], [27], [28], [36].

Omega-3 fatty acid supplements induce production of less immunosuppressive types of prostaglandin [37]. Omega-3 fatty acids inhibit in-vitro proliferation of lymphocytes but prolong the survival of dormant lymphocytes in the cell culture [38]. Fatty acid diets also influence the production of cytokines, especially of IL-1, IL-6, IL-2 and TNF-α [39]. Of the 260 patients awaiting pancreaticoduodenectomy or gastrectomy, 87 received a standard diet, another 87 received immunonutrition containing arginine, omega-3 fatty acids and nucleotides, and 86 patients received total parenteral nutrition [40]. On the 8^th^ postoperative day, the patients receiving immunonutrition showed a significantly better recovery of their immunological parameters in comparison to the other groups [40]. Inverse correlation of IL-6 with prealbumin was observed only in this group. The postoperative infection rate was 14.9% in immunonutrition patients, 22.9% in standard diet patients, and 27.9% in parenteral nutrition patients. Similarly, the length of hospital stay was 16.1±6.2, 19.2±7.9, and 21.6±8.9 days [40]. In a different study involving 60 cancer patients, supplementation with omega-3 fatty acids led to an increase in the CD4/CD8 ratio and TNF-α production [27]. In an animal experiment on rats, supplementation with long-chain fatty acids increased the cytotoxicity of NK cells and raised the amounts of activated T and B lymphocytes as well as monocytes following stimulation with Con A [41]. Supplementation with arginine, omega-3 fatty acids, and nucleotides resulted in significantly increased DR expression on monocytes (improves antigen presentation) on the 7^th^ postoperative day in 16 patients with severe traumas and in the clinical course led to fewer days with systemic inflammatory response syndrome during the first 28 postoperative days compared with patients with severe traumas without immunonutrition (n=13) [42].

## 3 Objectives

The clinical trial was carried out in cooperation with intensive care physicians, ENT surgeons and experimental immunologists and was designed to answer the following questions:

1. Can the length of hospital stay and the prognosis of multiple trauma patients and patients after extensive ENT surgery be improved by early enteral nutrition with specific basic nutrients?
2. What effect does early enteral nutrition therapy have on wound healing?
3. Can a nutritional intervention have immunostimulating effects?
4. Can the length of inpatient stay (ICU and general wards) be reduced by early enteral nutrition and suitable diets?

In this research project, patients with extensive surgery or multiple trauma were to be provided with combined enteral nutrition specifically tailored to the metabolic conditions of this patient group.

Several immunological parameters were to be monitored by the analysis of blood samples taken directly before treatment (preoperative) as well as 1 day, 3 days, 5 days, 7 days, 10 days and 14 days after treatment. In addition, factors such as wound healing, consumption of antibiotics, and incidence of septic complications were to be analysed during the ICU stay and total hospital stay.

### 3.1 Working hypothesis

The benefit of glutamine for the intestinal mucosa and the prognosis of surgical as well as multiple trauma patients is well documented for parenteral applications. Infectious complications such as bacterial translocation and mucosal atrophy as well as increasing budgetary pressure necessitate early enteral supplementation with glutamine and omega-3 fatty acids. This project was designed to determine whether similar benefits can be achieved by means of an enteral nutrition regimen.

- When it is initiated within the first 24 hours after trauma, a glutamine-enhanced enteral diet will lessen infectious complications, improve wound healing, and reduce the length of stay in the ICU as well as the total hospital stay.

### 3.2 Objective criteria

#### 3.2.1 Primary objective criteria

Shortened inpatient stay (ICU and general wards)

#### 3.2.2 Secondary objective criteria

**a)** **Immune response:** measurement and observation of the following variables:

- cytokines: sIL-1RA, sIL2R, IL-2, IL-3, IL-4, IL-6, sIL-6R, IL-10, TNF-α IFN-γ, TGF-β2, neopterine
- specific infection parameters: CRP
- lymphocyte differentiation: total lymphocyte count, CD4/CD8 ratio, CD3+, CD4+, CD8+, CD16+, CD19+, CD3+DR+, CD3+CD25+, CD4+DR+, CD8+DR+
- lymphocyte transformation tests with PWM, PHA, Con A, CD3 mAk, MLC with pooled allogeneic stimulator cells
- infectious complications (especially pneumonia, sepsis, wound infection, urogenital tract)
- i.v. antibiosis (days), dosage; number of ventilation days

**b)** **Nutritional state:** BMI, body fat measurement 

**c)** **Safety:** clinical laboratory, especially liver enzyme, creatinine, electrolyte levels, additionally albumin, immune globulin

**d)** **Tolerability:** diarrhoea, vomiting, obstipation, meteorism

**Assessment of clinical situation:** ISS, GCS, APACHE 11, MOF, SOFA, SAPS

## 4 Methods

### 4.1 Immune response

The immune response is characterised by the relative percentages and numbers of all lymphocyte subpopulations involved in immune response, by the activation level of individual cell populations, by the functionality of lymphocytes (tested under laboratory conditions), and the blood levels of soluble mediators with a stimulating or inhibiting effect on immune response such as cytokines, soluble cytokine receptors, soluble cytokine antagonists, and soluble adhesion molecules. 

### 4.2 Assessment of immunological factors 

Immunological tests were carried out in the Transplantation Immunology Department at the Institute of Immunology of the Heidelberg University. The immunological tests described below have been established for years and have been described in numerous international publications.

#### 4.2.1 Material

- 30 ml heparin blood for the assessment of lymphocyte subpopulations and to carry out the lymphocyte transformation test
- 5 ml plasma for cytokines, soluble cytokine receptors and antagonists, soluble adhesion molecules and neopterin

The heparin blood samples were immediately transferred to Heidelberg; the plasma samples were frozen in Ulm and transported to Heidelberg on dry ice.

#### 4.2.2 Assessment of lymphocyte subpopulations

Three-colour flow cytometry was used to determine the percentages and absolute cell counts of CD3+ T lymphocytes, CD4+ T helper cells, CD8+ suppressor/cytotoxic T lymphocytes, CD16+ NK cells and CD19+ B lymphocytes. Further tests determined the percentages and counts of IL-2 receptor-bearing (CD25+) activated CD4+ and CD8+ T lymphocytes as well as DR+ activated CD4+ and CD8+ T lymphocytes.

A deficit in CD4+ T helper cells means immune suppression, and a surplus of CD8+ suppressor/cytotoxic T lymphocytes suggests an ongoing immune response to an antigen, e.g. a virus. IL-2 receptors and DR antigens on the cell membrane of T lymphocytes indicate the activation levels of individual lymphocyte subpopulations. 

#### 4.2.3 Lymphocyte transformation test with mitogens and/or pooled allogeneic stimulator cells

Mononuclear cells are isolated from heparin blood by Ficoll density gradient centrifugation. Subsequently, 10^5^ (10^6^/ml) lymphocytes per well are incubated for 3 days in triple culture with the mitogens concanavalin A, pokeweed mitogen, phytohaemagglutinin or anti-CD3 monoclonal antibody. Each mitogen is tested in three different concentrations. In addition, patient lymphocytes are incubated for 6 days in mixed lymphocyte culture with irradiated pooled allogeneic stimulator cells. The cells are then pulsed with ^3^H thymidine for 18 hours and harvested and a beta counter is used to measure the amount of ^3^H thymidine incorporated into the genome. 

Patient results are compared with the results of a healthy control person tested at the same time. Relative stimulation indices are calculated to compare test cultures carried out on different days.

The lymphocyte transformation test described above analyses three different stimulation approaches, i.e. cell activation by membrane-bound (a) mitogen receptors, (b) CD3 determinants and (c) T cell receptors.

Insufficient in vitro response suggests an in vivo dysfunction of T lymphocytes, especially T helper lymphocytes.

#### 4.2.4 Cytokines, soluble cytokine receptors and cytokine receptor antagonists, soluble adhesion molecules, neopterin

ELISA tests are performed to analyse plasma levels of soluble interleukin-1 receptor antagonist (sIL-1RA), IL-2, sIL-2R, IL-3, IL-4, IL-6, sIL-6R, IL-10, tumour necrosis factor-α (TNF-α), transforming growth factor-β_2_ and interferon-γ (IFN-γ) as well as neopterin. 

Changes in plasma levels are indications of:

- impaired monocyte/macrophage/T helper lymphocyte interaction (increase in monokines and lack of lymphokines)
- activation of TH1 (induces cellular defence) or TH2 lymphocytes (induces humoral defence, has immunosuppressive effects on cellular defence)
- induction of immunosuppressive cytokines (e.g. TGF-β_2_), cytokine receptor antagonists and soluble cytokine receptors (competition with corresponding immunostimulating cytokine).

By using this test panel we obtained data on the numerical distribution and activation level of individual lymphocyte subpopulations involved in the immune response. The lymphocyte transformation test established lymphocyte functionality. The analysis of plasma levels of soluble mediators allowed us to draw conclusions about the cause of immunosuppression, in other words about the impaired functionality of immune response.

## 5 Results

All in all, 14 patients with extensive surgery (8 with glutamine-supplemented tube nutrition/6 with isonitrogenous nutrition) and 7 multiple trauma patients (4 with glutamine-supplemented nutrition/3 with isonitrogenous nutrition) were examined.

**Figure 1:**
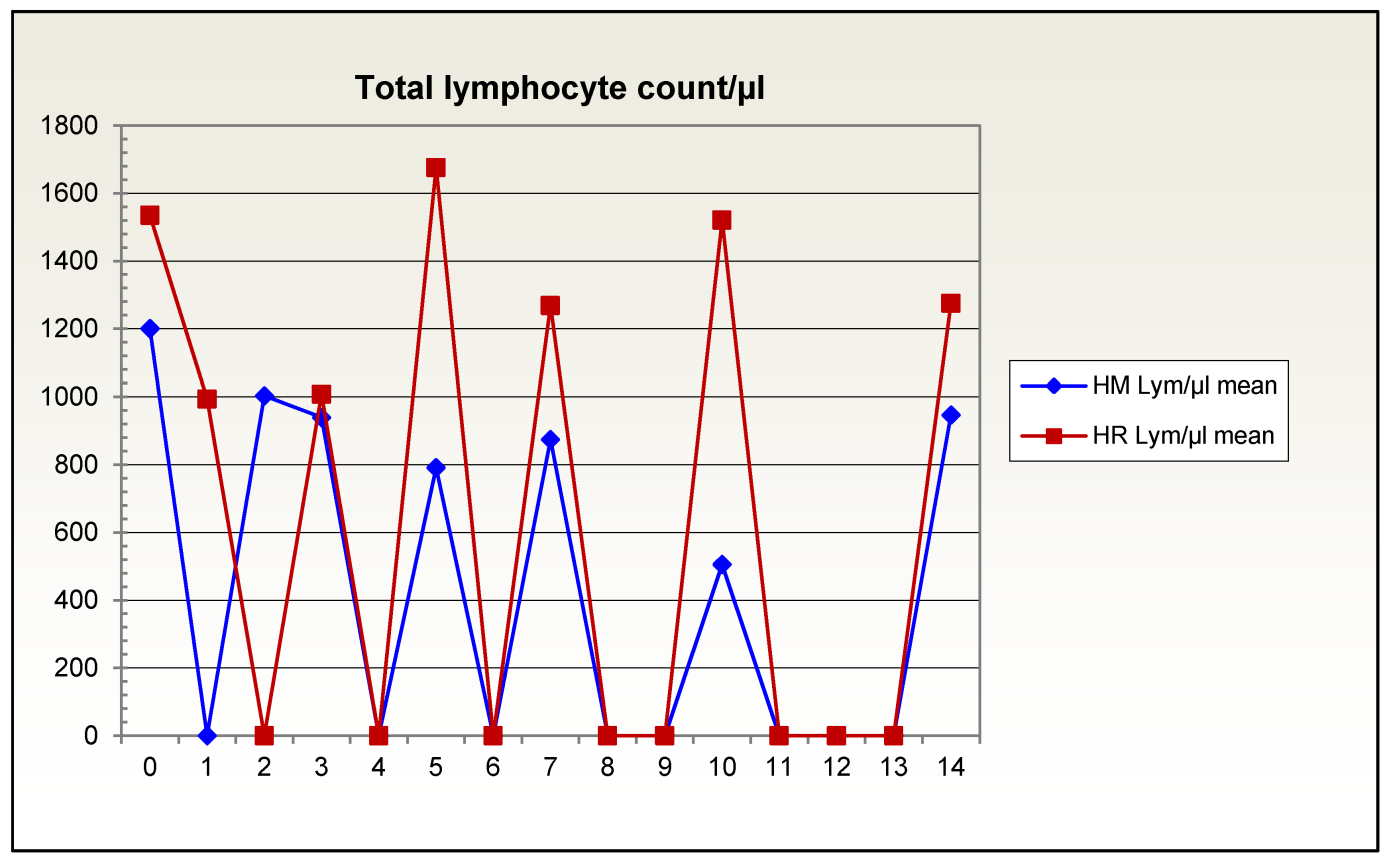
Total lymphocyte count over 14 days in patients given glutamine-supplemented (HR) versus isonitrogenous nutrition (HM)

In patients fed with Reconvan (HR in Figure 1 (Fig. 1)), the total lymphocyte count increased from the third day of feeding, whereas in patients with isocaloric, isonitrogenous nutrition (HM in Figure 1 (Fig. 1)) lymphocyte counts fell and remained constant at low levels (Figure 1 (Fig. 1)).

**Figure 2:**
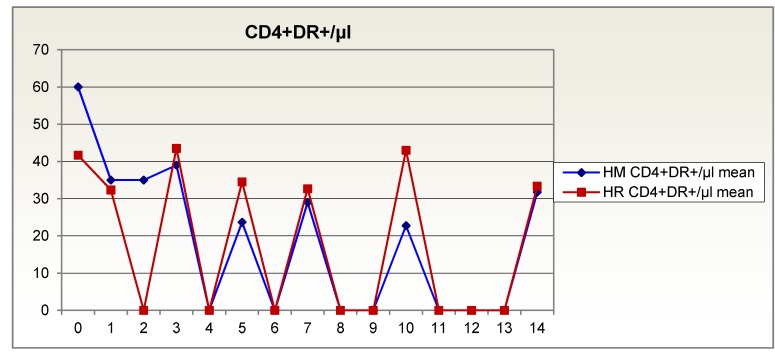
Activated CD4+DR+ T helper lymphocytes over 14 days in patients given glutamine-supplemented (HR (red)) versus isonitrogenous nutrition (HM (blue))

Patients fed with Reconvan showed an increase in activated CD4+DR+ T helper lymphocytes. Their blood level of CD4+DR+ lymphocytes remained above 30%. The isonitrogenous group, however, remained at a low level after the posttraumatic decline in activated CD4+DR+ T helper lymphocytes and during the first two postoperative weeks invariably had lower mean values than the Reconvan patients (Figure 2 (Fig. 2)).

**Figure 3:**
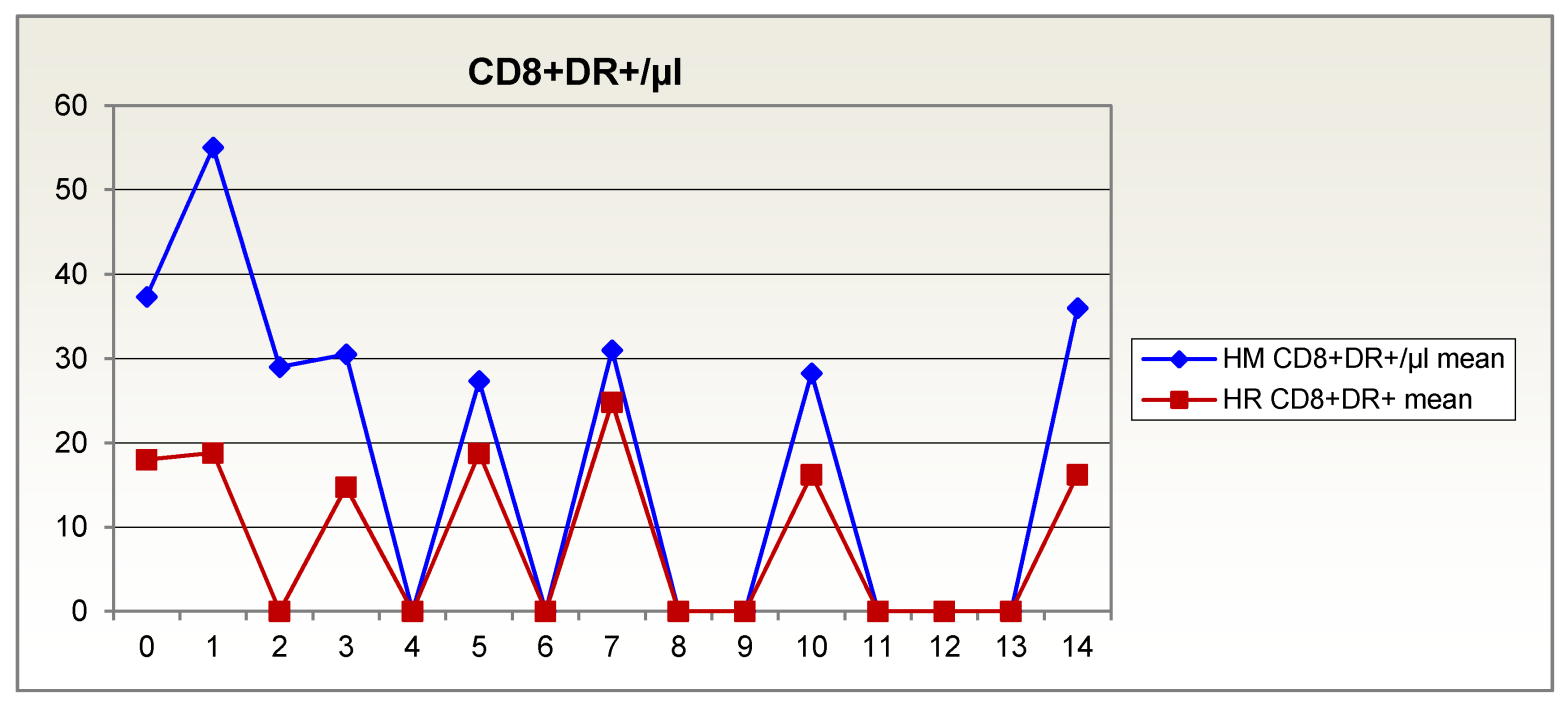
Activated CD8+DR+ suppressor cells/cytotoxic T lymphocytes over 14 days in patients given glutamine-supplemented (HR (red)) versus isonitrogenous nutrition (HM (blue))

The opposite is true for the activated CD8+DR+ suppressor cells/cytotoxic T lymphocytes. The supplemented group remained at a lower level than the isonitrogenous group (Figure 3 (Fig. 3)).

**Figure 4:**
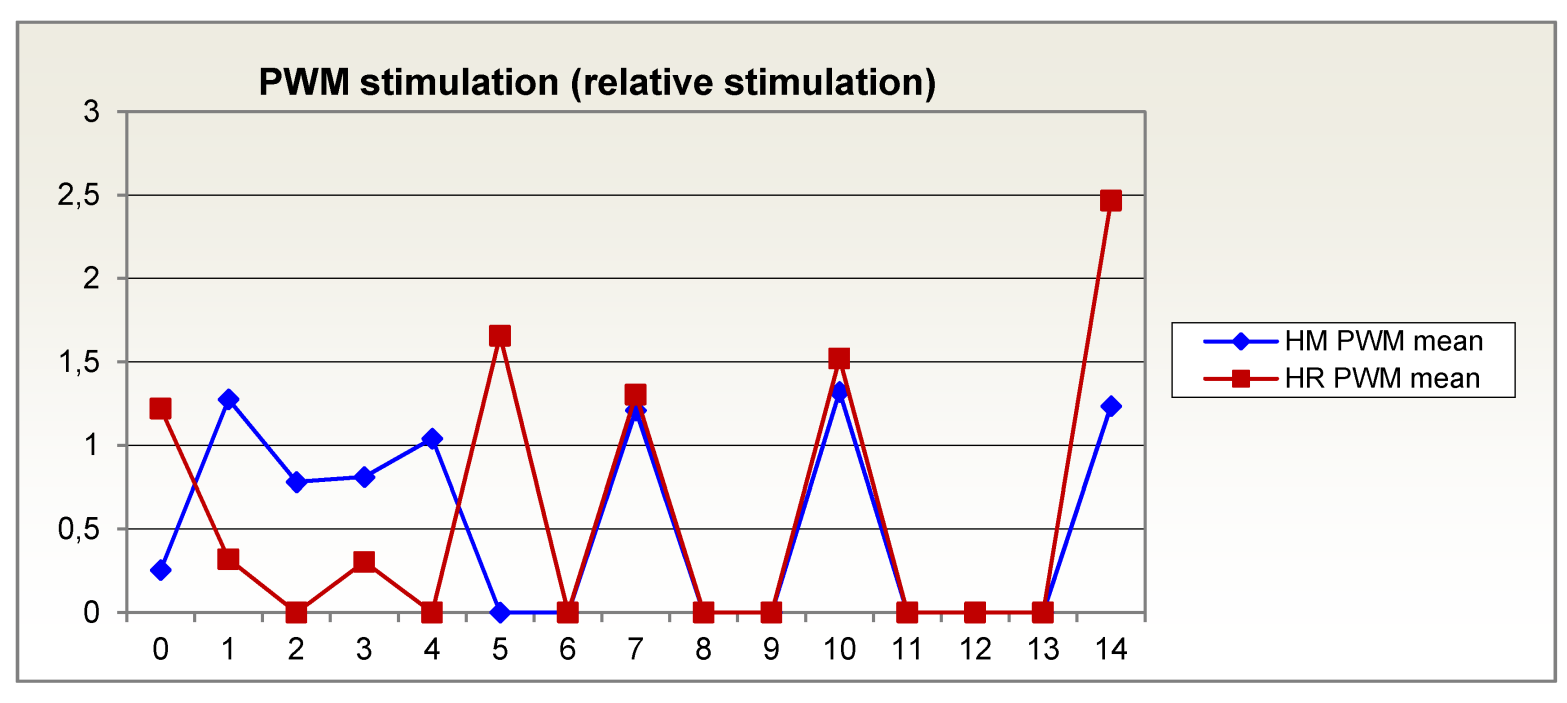
In vitro response of lymphocytes, measured as relative stimulation with pokeweed mitogen, over 14 days in patients given glutamine-supplemented (HR (red)) versus isonitrogenous nutrition (HM (blue))

**Figure 5:**
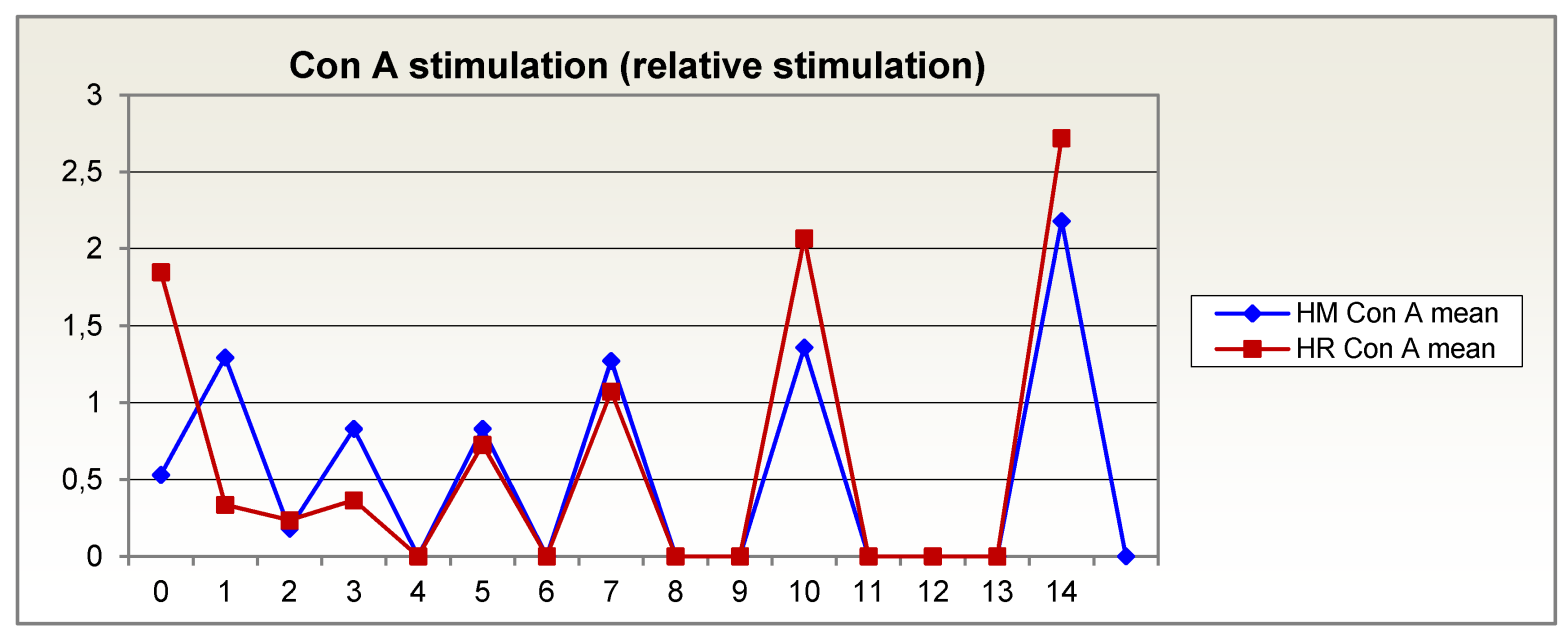
In vitro response of lymphocytes, measured as relative stimulation with concanavalin A, over 14 days in patients given glutamine-supplemented (HR (red)) versus isonitrogenous nutrition (HM (blue))

In the first days after trauma, the in vitro response of lymphocytes, measured as relative stimulation with concanavalin A and pokeweed mitogen, was much worse for the glutamine-supplemented group than for the isocaloric, isonitrogenous group. Lymphocyte response recovers sooner in Reconvan patients than in patients on an isonitrogenous diet. During the second week after trauma, it was on average higher in Reconvan patients than in the control group. At the end of the second week after trauma, it was even higher than in control persons tested at the same time (normal: relative stimulation = 1) (Figure 4 (Fig. 4), Figure 5 (Fig. 5)).

**Figure 6:**
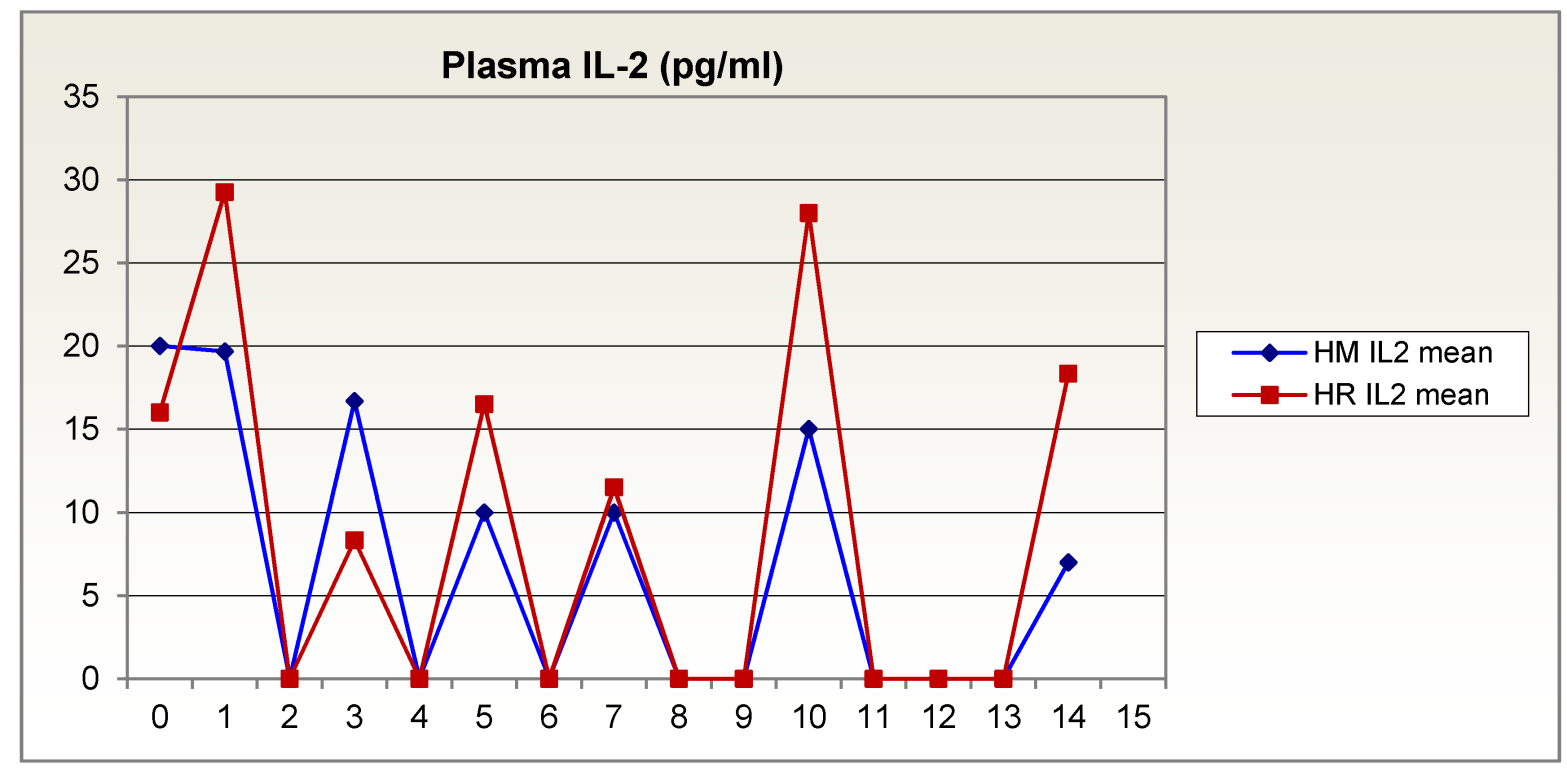
Interleukin-2 plasma levels over 14 days in patients given glutamine-supplemented (HR (red)) versus isonitrogenous nutrition (HM (blue))

Interleukin-2 plasma levels, too, recovered much sooner in the glutamine-supplemented group than in the isocaloric, isonitrogenous group (Figure 6 (Fig. 6)). In the second week after trauma, some values exceeded the normal range of 0–20 pg/ml in healthy individuals. 

The clinical parameters were as follows:

- **Wound healing problems:** The glutamine-supplemented group had fewer wound healing problems (0/12) than the isocaloric, isonitrogenous group (3/9).
- **Infections:** As far as infectious complications are concerned, the glutamine-supplemented group had no infections and thus did much better than the isocaloric, isonitrogenous group, which had 3 infectious complications (2 cases of pneumonia, 1 case of sinusitis).

## 6 Discussion

A number of studies have confirmed the advantages of enteral nutrition over total parenteral nutrition in critically ill patients. Major advantages include maintenance of the gastrointestinal function and stress ulcer prevention. Moreover, enteral nutrition helps to improve the gastrointestinal function and thus to support regional defence mechanisms and ultimately to strengthen the immune system [3], [4], [7], [27], [30], [43]. For example, burn patients and patients after surgery for malignant neoplasms who received enteral nutrition therapy had lower complication rates and shorter hospital stays [33], [35]. In addition to the type of nutrition, the beginning of nutritional measures is also of major significance [26]. Chiarelle et al., who compared burned patients who received early and late nutritional therapy, found a decrease in positive blood cultures and a shortened hospital stay in the early nutrition group [44]. Bauer et al. observed a reduction in mortality in ICU patients who received early enteral nutrition [45]. Our results appear to support the hypothesis that early glutamine-supplemented enteral nutrition compared with isocaloric, isonitrogenous nutrition has positive effects on the immune system and the course of disease in ICU patients. Immunologically, early glutamine-supplemented enteral nutrition compared with isocaloric, isonitrogenous nutrition appears to accelerate the stabilisation of posttraumatic immunosuppression. This is reflected in the consolidation of total lymphocyte counts and – for the subpopulations – in that of the T helper cells and T suppressor cells. It is important to note that lymphocytes take a lead role in cellular defence. T lymphocytes are linked via cytokines with all immune structures and thus regulate the immune response. They can strengthen immune response as CD4+ helper cells, and they can weaken immune response as CD8+ T suppressor cells. Following antigen presentation, T helper cells stimulate the production of antibodies in B cells. In addition, they coordinate lymphocyte interaction and are highly capable of interacting with macrophages. These functions make CD4+ cells a critical element of the immune system. This is why their dysfunction can have a major impact on the appropriate response of the organism to pathogens. Lymphocyte dysfunction and destabilisation of total lymphocyte as well as CD3+ and CD4+ cell counts could be the immunological correlate of the number of septic complications in multiple trauma patients. Our findings underline the influence of nutrition on the immune system. Where possible, multiple trauma patients and patients with extensive surgery should be given early glutamine-supplemented enteral nutrition. This is often impeded by gastroparesis and reflux in this group of patients. This problem can be addressed by a tube in the distal duodenum and jejunum since the small intestine is able to resume its function soon after trauma. Problems with diarrhoea are extremely rare. As a result of gastric decompensation, undesired side effects such as reflux and vomiting can be avoided.

The findings obtained so far appear to confirm the hypothesis of a positive influence on the immune system in critically ill patients. It is possible that this hypothesis also applies to clinical parameters such as the length of hospital stay and the incidence of septic complications, but this must be confirmed in a larger patient sample. If we consider the lessons learned by other teams who have dealt with this problem, we can assume that the average infection rate in intensive care units (pneumonia, sepsis, sinusitis, urinary tract infections) is approximately 45% (46% in the author’s patient population). Houdjik and Ziegler achieved the following results with a glutamine-supplemented diet in comparable patient groups [11], [46] (see Table 1 (Tab. 1)).

In summary, the preliminary data of our interim analysis show that total lymphocyte counts, the percentage of activated CD4+DR+ T helper lymphocytes, the in-vitro response of lymphocytes with mitogens, as well as IL-2 plasma levels normalise faster in patients who receive glutamine-supplemented nutrition than in patients who receive isocaloric, isonitrogenous nutrition and that these parameters are even above normal by the end of the second postoperative week.

Enteral nutrition treatment with glutamine is a cost-effective therapeutic approach that is easy to implement and that promises a marked improvement in prognoses, especially in patients with extensive soft tissue injuries resulting from trauma or surgery.

This type of therapy can also be applied in crisis areas.

## Notes

### Competing interests

The authors declare that they have no competing interests.

## Figures and Tables

**Table 1:**
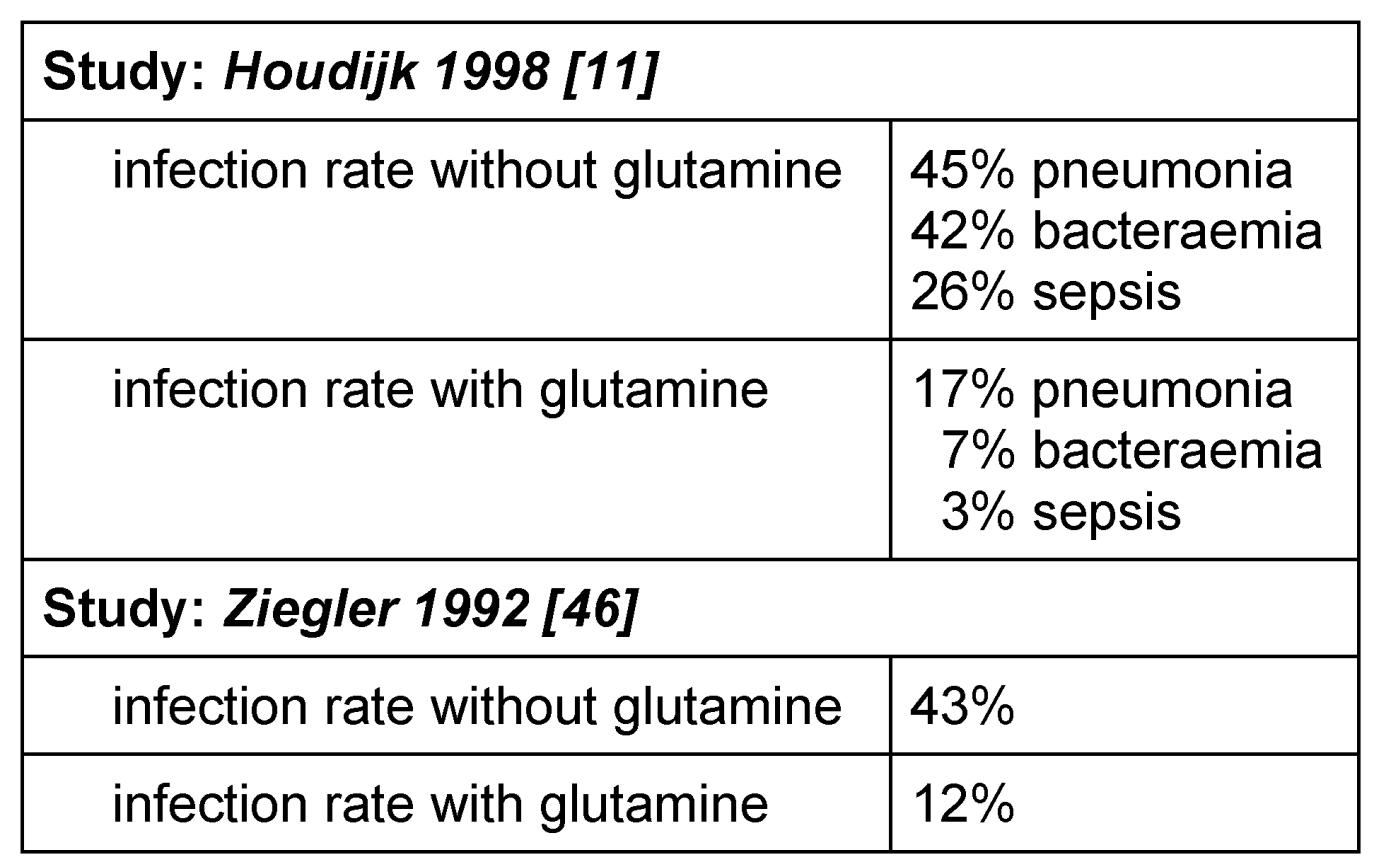
Infection rates of intensive care patients with and without glutamine supplements
